# Mutations in the *CDSN* gene cause peeling skin disease and hypotrichosis simplex of the scalp

**DOI:** 10.1111/1346-8138.15136

**Published:** 2019-10-29

**Authors:** Jaap J. A. J. van der Velden, Michel van Geel, Jans J. Engelhart, Marcel F. Jonkman, Peter M. Steijlen

**Affiliations:** ^1^ Department of Dermatology Maastricht University Medical Center+ Maastricht The Netherlands; ^2^ GROW Research School for Oncology and Developmental Biology Maastricht University Medical Center+ Maastricht The Netherlands; ^3^ Department of Dermatology Ommelander Ziekenhuis Groep Delfzijl; ^4^ Department of Dermatology University of Groningen University Medical Center Groningen Groningen The Netherlands

**Keywords:** corneodesmosin, genetic, hypotrichosis simplex of the scalp, peeling skin disease, skin diseases

## Abstract

Peeling skin disease is a rare genodermatosis characterized by superficial exfoliation or peeling of the skin. Peeling skin disease is caused by biallelic mutations in *CDSN* as an autosomal recessive trait. Monoallelic mutations in *CDSN* have also been described in an autosomal dominant inherited genodermatosis: hypotrichosis simplex of the scalp. This disease is characterized by progressive hair loss of the scalp with onset after early childhood. Clinical data were obtained from a patient with lifelong generalized skin peeling and both his parents. The patient's parents did not suffer from skin peeling, but the mother had a history of thin scalp hair since early childhood. Mutation analysis in the patient showed compound heterozygous mutations in exon 2 of *CDSN*, a nonsense mutation c.598C>T (p.[Gln200*]), previously associated with hypotrichosis simplex of the scalp, and a frame‐shift mutation c.164_167dup (p.[Thr57Profs*6]), previously described in peeling skin disease. The p.(Gln200*) mutation was also found in the mother of the proband. Our study strengthens the previously established link between mutations in *CDSN* to peeling skin disease and hypotrichosis simplex of the scalp.

## Introduction

Peeling skin syndrome (PSS) was first described by Fox in 1921.^1^ PSS is a rare autosomal recessive genodermatosis characterized by superficial exfoliation or peeling of the skin. It is classified into localized and generalized forms. Acral peeling skin syndrome (APSS; PSS2; Mendelian Inheritance in Man [MIM] #609796) is considered a localized variant of PSS and is caused by mutations in *TGM5* encoding transglutaminase 5 (TGM5).^2^ The generalized form is subclassified into non‐inflammatory (type A) (PSS3; MIM #616265) and inflammatory (type B) forms (PSD; PSS1; MIM #270300).^3^ PSS type A is linked to mutations in *CHST8*.^4^ Recently, mutations in *FLG2* were described in patients with generalized skin peeling.^5^, ^6^

In PSS type B, also referred to as peeling skin disease (PSD), mutations in *CDSN* 7, 8, 9, 10, 11, 12, 13 and large deletions encompassing *CDSN* 14 have been described. Corneodesmosin (CDSN) is expressed in hair follicles and cornified epithelia and is considered to play an important role in cell–cell adhesion.^15^ PSD is characterized by congenital ichthyosiform erythroderma with recurrent superficial exfoliation or peeling of the skin. It is accompanied by pruritus and (other) atopic manifestations, mostly accompanied by high levels of immunoglobulin (Ig)E.^16^

Histological and ultrastructural analyses demonstrate that cleavage occurs immediately above the stratum granulosum or within the stratum corneum with overlying compact or basket weave hyperkeratosis.^7^ We report a case of PSD caused by compound heterozygous mutations in *CDSN*.

## Methods

All the studies described were conducted in accordance with the Declaration of Helsinki with ethical approval and informed consent.

### Patient

**Figure 1:**
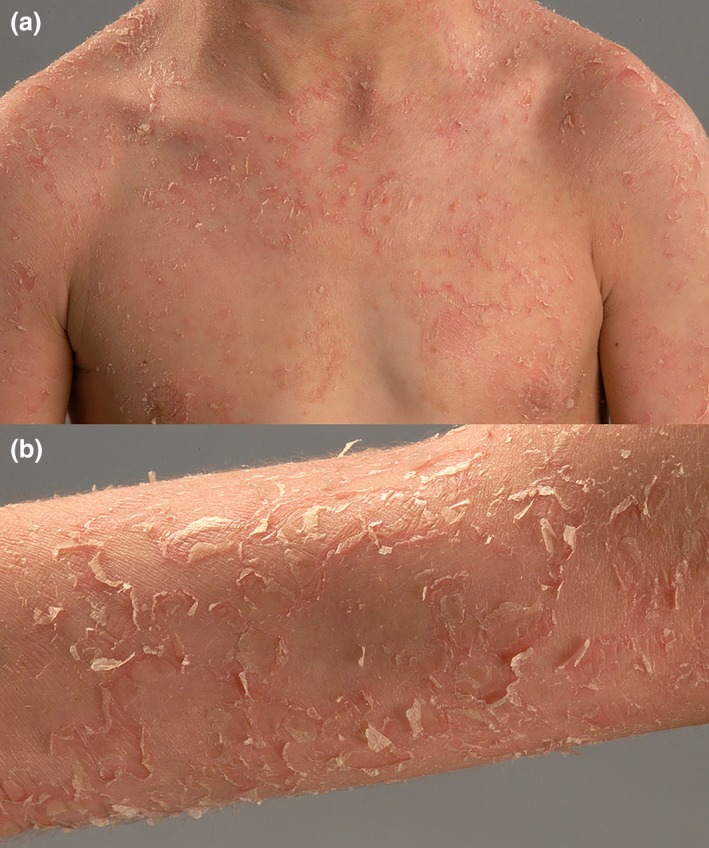
Index patient with generalized superficial skin exfoliation/blistering with partly erythematous background. (a) Overview, (b) detail.

A 19‐year‐old man visited our outpatient clinic in 2011. From the age of 2 years, he had visited multiple dermatologists in other clinics. He suffered from pruritic sub‐erythroderma with extensive skin peeling and scaling since birth (Fig. 1). The condition tended to be worse in winter. He had complaints of atopic dermatitis and hay fever. Hair, teeth and nails appeared normal. Routine laboratory parameters were normal, except for elevated IgE levels (2222 kU/L; normal, <100). Hair shaft analysis did not show trichorrhexis invaginata. As the phenotype resembled Netherton syndrome, mutation analysis of *SPINK5* was performed: no pathogenic mutations were detected. Over the years, the patient was treated with topical moisturizing agents, local corticosteroids and a prolonged course of low‐dose isotretinoin; only the latter improved complaints slightly according to the patient.

**Figure 2:**
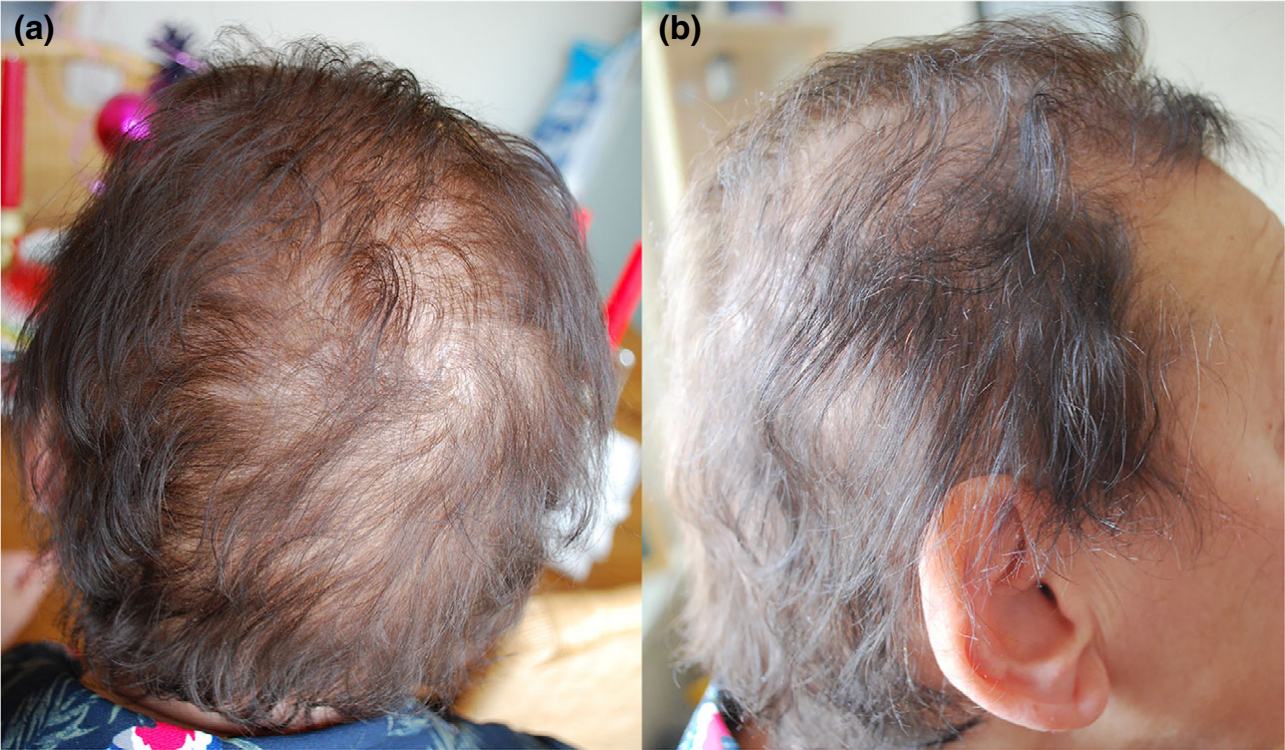
Mother of the index patient showed diminished hair density on the scalp. (a) Dorsal view, (b) side view.

**Figure 3:**
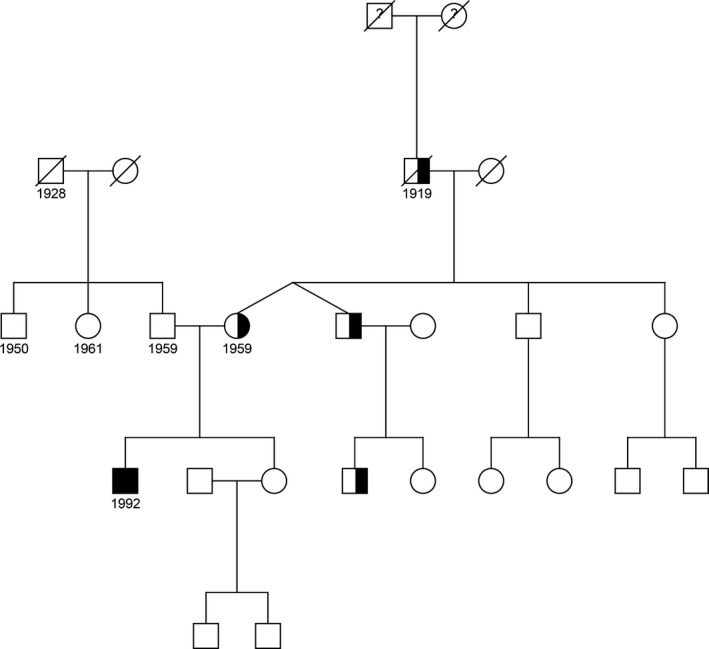
Family pedigree. The filled symbol depicts the proband with skin peeling and without hypotrichosis; the partially filled symbols depict subjects with hypotrichosis, birth year displayed underneath the symbols if known.

His non‐consanguineous parents of Lithuanian and Russian Jewish descent did not show any skin symptoms. Initially, the proband stated that his parents had no history of obvious hair loss. After obtaining the results of the mutation analysis, the mother was assessed in our outpatient clinic. The mother had a lifelong history of thin scalp hair, gradually worsening as she aged. Upon examination, the diagnosis hypotrichosis simplex of the scalp (HSS) was made (Fig. 2). According to the mother, her twin brother and his son and her father shared the same hair phenotype (Fig. 3).

### Skin tissue analysis

Histopathology (hematoxylin–eosin staining) of a skin biopsy showed compact hyperkeratosis with focal parakeratosis and acanthosis with basal separation in the stratum corneum and a subepidermal lymphocytic perivascular infiltrate.

Electron microscopy showed granular/fibrillar degeneration of the upper corneocytes with intact cell membranes. Intracorneal cleavage with intercellular deposition of fibrillar material was seen. Corneodesmosomes appeared normal. Normal keratohyalin granules were observed in the stratum granulosum. In the papillary dermis, multiple round amorphic amyloid bodies were seen (Supporting information).

### Mutation analysis

Genomic DNA from the affected individual and parents was isolated from peripheral blood leukocytes using the QIAampTM blood kit (Qiagen, Hilden, Germany). The two coding exons and adjacent splice sites of *CDSN* were amplified by polymerase chain reaction using primers as previously described.^7^ Automated DNA sequencing analysis was performed on an ABI Prism 3730 genetic analyzer (Applied Biosystems, Foster City, CA, USA).

## Results

Mutation analysis in our patient showed two heterozygous mutations in exon 2 of *CDSN*, designated c.598C>T and c.164_167dup (NCBI reference sequence: NM_001264.4). The mother was heterozygous for the c.598C>T mutation, whereas the father was heterozygous for the c.164_167dup mutation (Supporting information).

The c.598C>T mutation putatively results in a premature stop codon p.(Gln200*) in the corneodesmosin protein. This mutation was previously described in patients with HSS (MIM #146520).^17^ Amyloid aggregates of mutated corneodesmosin are found in hair follicles of these patients.^18^

The c.164_167dup mutation was previously described in patients with PSD and predicts a frame‐shift followed by a stop, p.(Thr57Profs*6).^10^ In patients with this mutation, pathogenicity was demonstrated by the absence of corneodesmosin expression in the granular layers in skin biopsies.^10^

## Discussion

**Figure 4:**
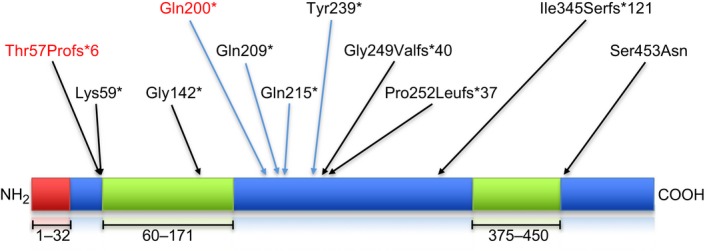
Schematic illustration of corneodesmosin and all known mutations. The glycine rich loops (amino acids 60‐171 and 375‐450) are marked green; the signal peptide (amino acids 1‐32) is marked red. All known mutations causing inflammatory peeling skin disease are designated by black arrows; the mutations causing hypotrichosis simplex of the scalp by blue arrows. The mutations described in this article are highlighted in red.

The link between PSD and corneodesmosin is well established. Multiple pathogenic mutations of *CDSN* (Fig. 4) have been described.^7^, ^8^, ^9^, ^10^, ^11^, ^12^, ^13^ These mutations are homozygous or compound heterozygous as the mode of inheritance is autosomal recessive. Large homozygous deletions of 59 kb in the 6p21.3 region encompassing *CDSN* are reported in Japanese patients. Although the deletion encompasses several other genes (*C6orf15*, *PSORS1C1*, *PSORS1C2*, *CCHCR1* and part of *TCF19*), these patients show a phenotype resembling that of PSD without hypotrichosis.^14^

Mutations in *CDSN* have also been described in HSS.^17^, ^19^, ^20^, ^21^ HSS is a rare autosomal dominant genodermatosis characterized by progressive loss of scalp hair. Patients with the heterozygous nonsense mutations p.(Gln200*), p.(Gln209*), p.(Gln215*) and p.(Tyr239*) show hypotrichosis; however, a PSD phenotype is not observed. It is hypothesized that aggregation of truncated CDSN protein into amyloid structures exerts a toxic effect on the hair follicles of the scalp. The absence of a PSD phenotype in these patients could be attributed to the presence of a normal allele and wild‐type protein.^18^

Only mutations in the region of amino acids 200–239 lead to hair loss. Caubet *et al*.^18^ have shown that truncated mutants of CDSN which bear the Gly/Ser‐rich domain (GS domain) abnormally accumulate at the periphery of hair follicles. They assemble into non‐toxic fibrils and toxic ring‐shaped oligomeric structures. The c.164_167dup mutation in the father resulted in a relatively small peptide lacking the GS domain which consequently did not assemble into the oligomeric form and therefore no hair loss was observed.

The toxic effect of the amyloid aggregates was likely occurring in a dominant negative manner with wild‐type and truncated mutant corneodesmosin polypeptides. If the mutation occurs in the region of amino acids 200–239, a toxic ring‐shaped oligomer is formed in the presence of wild‐type corneodesmosin; otherwise, a non‐toxic fibrillar structure is formed. It is likely wild‐type corneodesmosin acts as a modifying factor. One could hypothesize that oligomers are formed by linking truncated mutants to any form of corneodesmosin bearing the GS domain: truncated polypeptides or wild‐type protein.

The absence of wild‐type protein could explain why no hair phenotype was observed in the 19‐year‐old patient, although he carried a mutation in the 200–239 region. Interestingly, skin biopsies showed amyloid bodies in the papillary dermis of the patient. It is possible that these bodies only consisted of the fibrillar aggregates. However, there was no functional data to confirm this assumption.

Affected HSS individuals have normal hair in early childhood but experience progressive loss of scalp hair beginning in the middle of the first decade and almost complete baldness by the third decade. The body hair, beard, eyebrows, axillary hair, teeth and nails are normal.^17^ The severity of the hypotrichosis is variable. Almost complete loss of scalp hair is described in patients with the p.(Gln200*) mutation. However, the mother of the proband, also bearing this mutation, showed hypotrichosis but was not completely bald. This suggests incomplete or reduced penetrance of this mutation. This suggestion is supported by the notion that *CDSN* is extremely polymorphic^22^ and the different polymorphic alleles may modify disease penetrance.

We believe the diagnosis of HSS is underreported. The relatively mild hair loss in the mother of the proband never led to the diagnosis of HSS before. The diagnosis was only made after the mutation in *CDSN* was found. This exemplifies the importance of careful assessment (including family history‐taking) of patients with relatively mild hypotrichosis. In these patients, mutation analysis of *CDSN* should be considered.

In this study, we emphasized the role of corneodesmosin in PSD and HSS. Functional corneodesmosin plays an important role in maintaining the integrity of the epidermal barrier.

## Conflict of Interest

None declared.

## Supporting information


**Figure S1.** Sequence chromatographs showing the mutations in exon 2 of *CDSN*, designated c.598C>T and c.164_167dup in the patient, father and mother.

**Figure S2.** Histopathology (hematoxylin–eosin staining) of a skin biopsy showing amyloid deposits in the papillary dermis.
