# Supplementary figures and images for: Mutations in the CDSN gene cause peeling skin disease and hypotrichosis simplex of the scalp
Source: J Dermatol. 2019 Oct 29;47(1):3–7. doi: 10.1111/1346-8138.15136 (PMC6973079; doi:10.1111/1346-8138.15136)

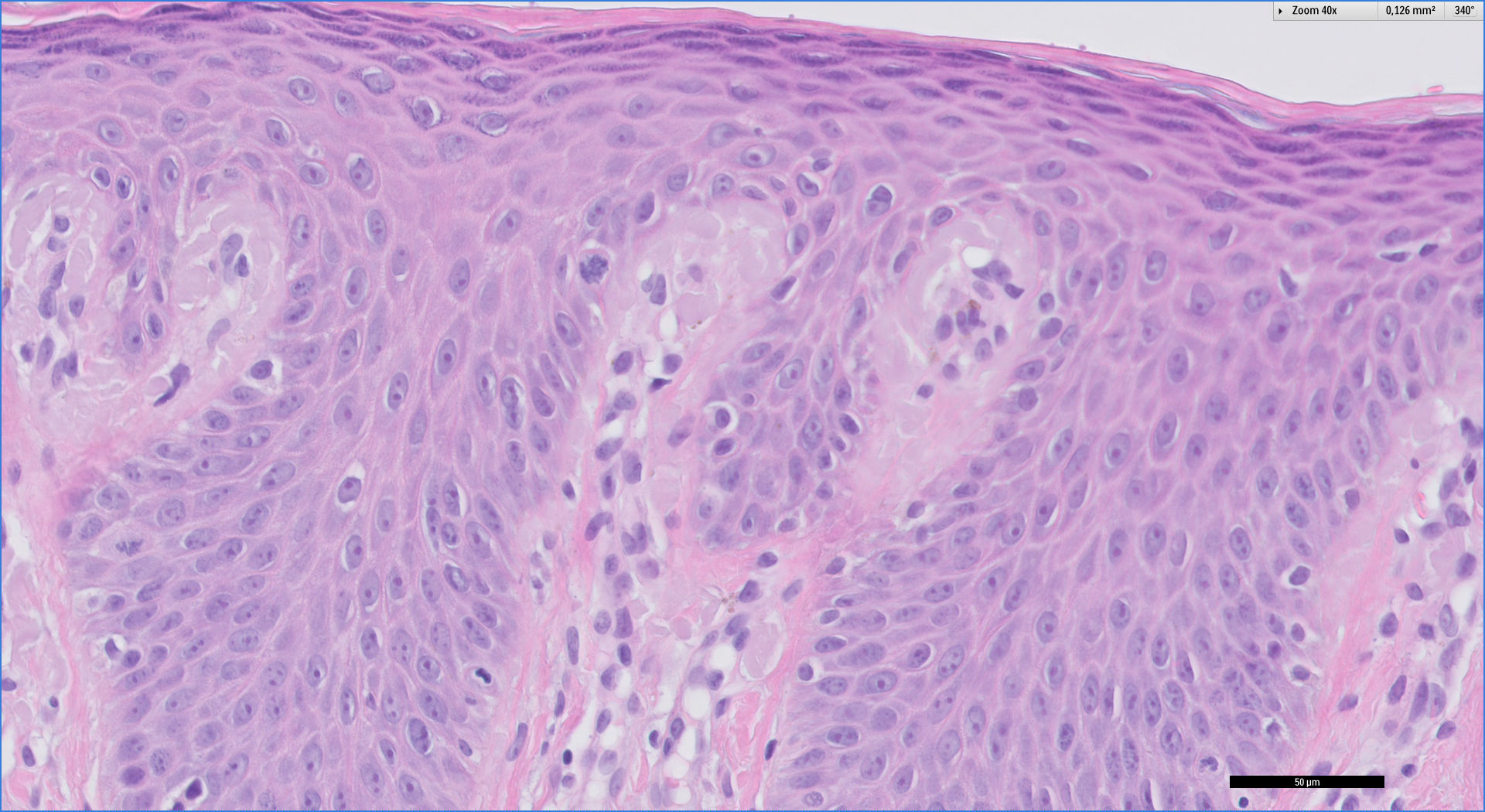

Supplement: Supplementary file 1 — Figure S1. Sequence chromatographs showing the mutations in exon 2 of CDSN, designated c.598C>T and c.164_167dup in the patient, father and mother. [file JDE-47-3-s001.jpg]

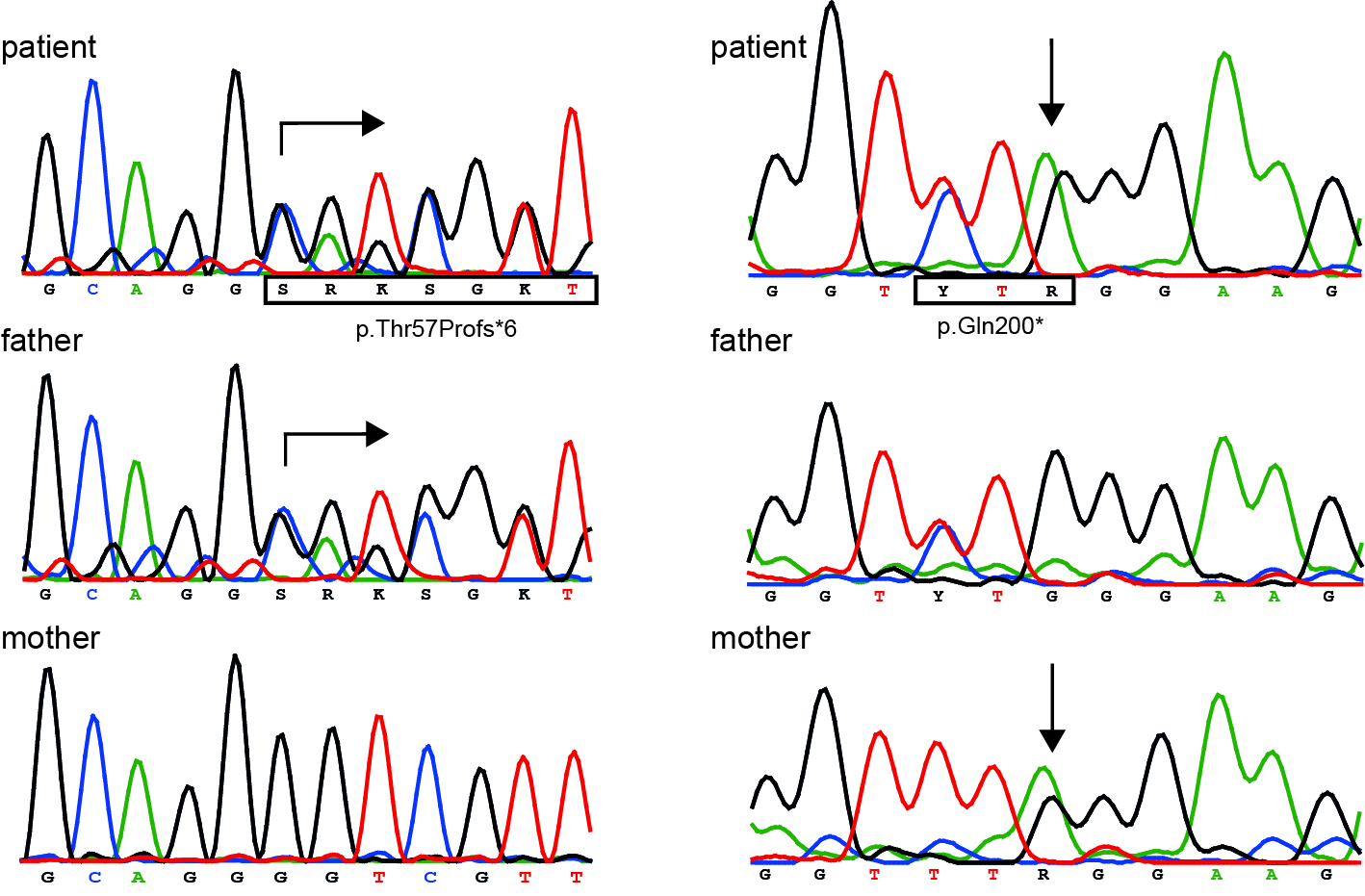

Supplement: Supplementary file 2 — Figure S2. Histopathology (hematoxylin–eosin staining) of a skin biopsy showing amyloid deposits in the papillary dermis. [file JDE-47-3-s002.jpg]
